# Supplementary figures and images for: Impact of secondary TCR engagement on the heterogeneity of pathogen-specific CD8+ T cell response during acute and chronic toxoplasmosis
Source: PLoS Pathog. 2022 Jun 21;18(6):e1010296. doi: 10.1371/journal.ppat.1010296 (PMC9249239; doi:10.1371/journal.ppat.1010296)

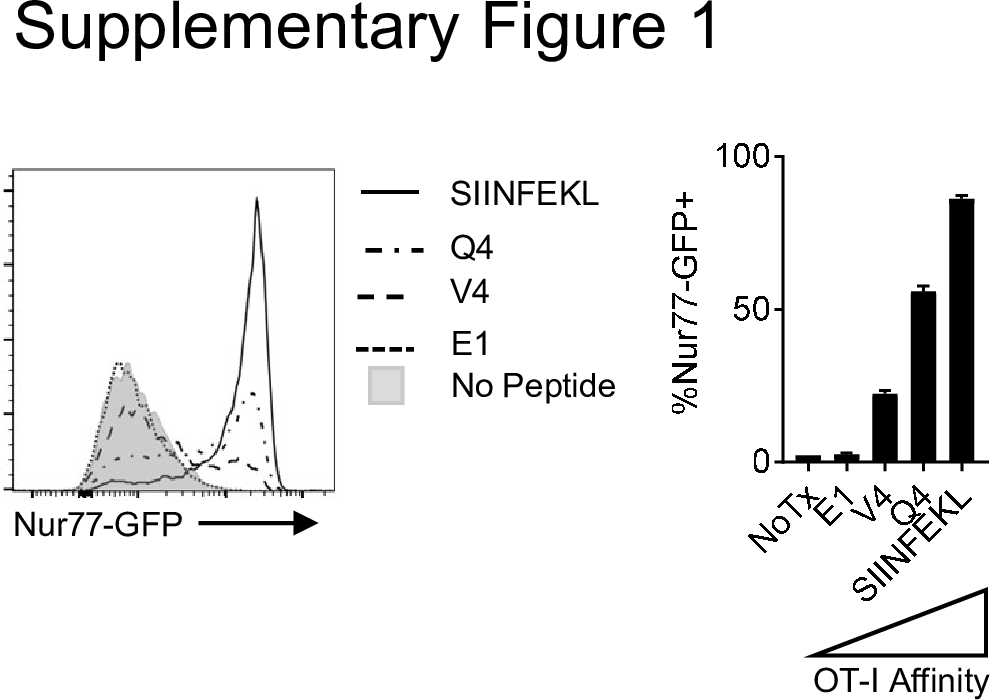

Supplement: S1 Fig — (A) Peptide pulsed splenocytes were incubated with Nur77-GFP OT-I and reporter activity was assessed at 3 hours (N = 2 technical replicates). (TIF) [file ppat.1010296.s002.tif]

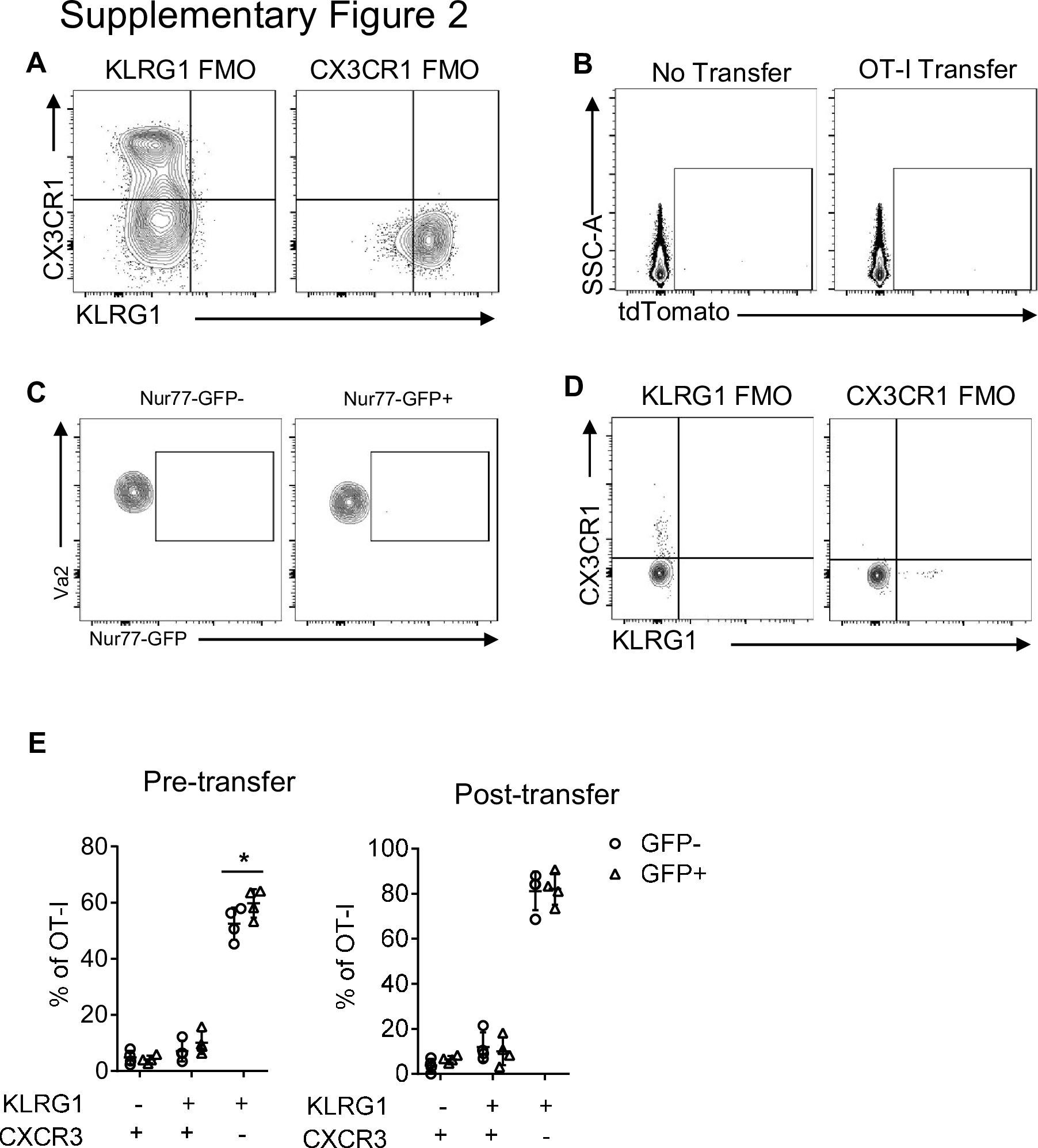

Supplement: S2 Fig — (A) FMO based gating for KLRG1 and CX3CR1 for Fig 3A. (B-E) OT-I from spleen of T. gondii-OVA 10 dpi were sorted by Nur77-GFP expression and transferred into naive mice. Mice were sacrificed 14 days post transfer (N = 4 mice). Lack of infection at 14 days post transfer measured by (B) tdTomato expression of total splenocytes or (C) Nur77-GFP expression in transferred OT-I. (D) FMO based gating for KLRG1 and CX3CR1 for Fig 3E. (E) KLRG1 and CXCR3 expression of Nur77-GFP- and Nur77-GFP+ OT-I prior to transfer at 10 dpi and 14 days post transfer. P values based on Two-Way ANOVA; error bars indicate SD. (TIF) [file ppat.1010296.s003.tif]

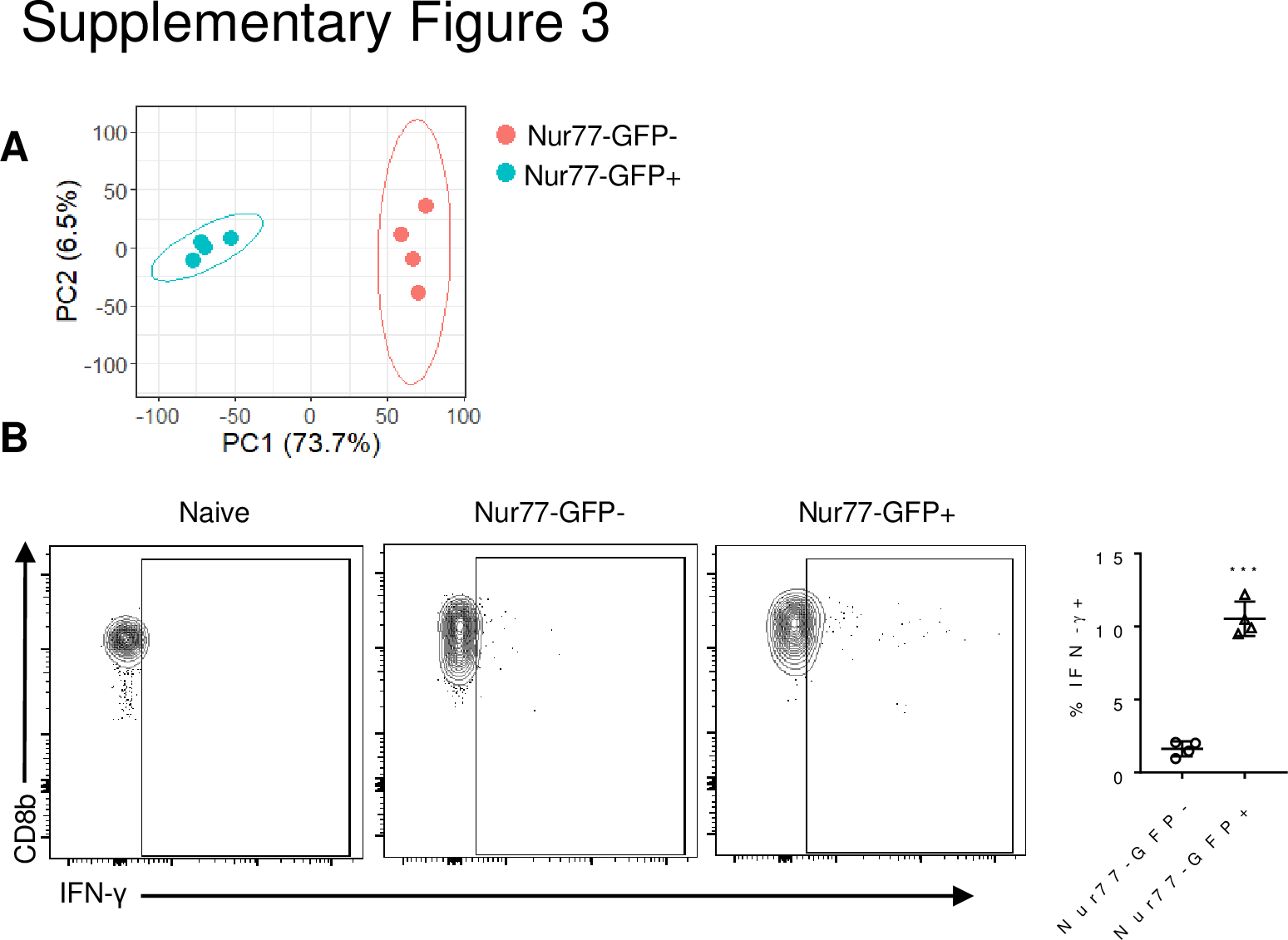

Supplement: S3 Fig — (A-B) Nur77-GFP- and Nur77-GFP+ OT-I from the brains of mice infected with T. gondii-OVA 14 dpi were transcriptionally profiled using RNA-sequencing (N = 4 pools of 2 mice per pool). (A) PCA plot of Nur77-GFP- and Nur77-GFP+ OT-I. (B) Endogenous cytokine production of Nur77-GFP- and Nur77-GFP+ OT-I from the CNS of T. gondii-OVA infected mice at 14 dpi. (TIF) [file ppat.1010296.s004.tif]

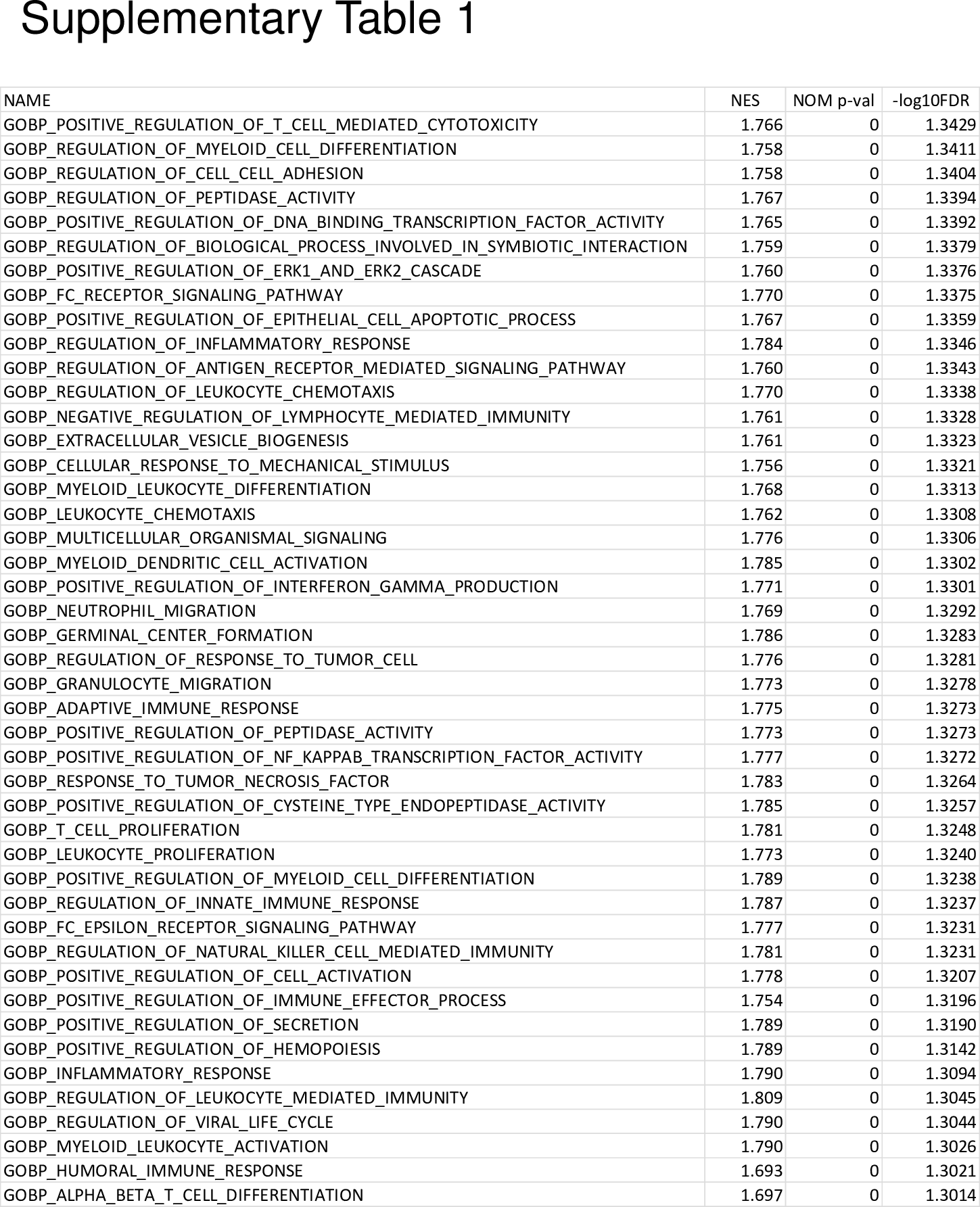

Supplement: S1 Table — Nur77-GFP- and Nur77-GFP+ OT-I from the brains of mice infected with T. gondii-OVA 14 dpi were transcriptionally profiled using RNA-sequencing (N = 4 pools of 2 mice per pool). Top GSEA gene sets with FDR>0.05 enriched in Nur77-GFP+ OT-I compared to Nur77-GFP- OT-I. (TIF) [file ppat.1010296.s006.tif]
