# Supplementary material for: Impact of secondary TCR engagement on the heterogeneity of pathogen-specific CD8+ T cell response during acute and chronic toxoplasmosis
Source: PLoS Pathog. 2022 Jun 21;18(6):e1010296. doi: 10.1371/journal.ppat.1010296 (PMC9249239; doi:10.1371/journal.ppat.1010296)
Supplement: Attachment — Submitted filename: Reviewer_response.docx [file ppat.1010296.s007.docx]

April 4th, 2022

The Editors,

Thank you for the opportunity to submit a revised version of the manuscript “Impact of secondary TCR engagement on the heterogeneity of pathogen-specific CD8^+^ T cell response during acute and chronic toxoplasmosis”. We appreciate the time and effort that went into these reviews and note the positive comments but also that they highlighted areas that required clarification. Consequently, we have modified several elements of the manuscript to address overarching questions about this model of infection and to clarify the literature that indicates that presentation of parasite antigen occurs within the CNS. Below are the individual responses to each specific comment and we thank the reviewers for help in improving the submission.

We hope that with these revisions and clarifications that this manuscript is now acceptable for publication in Plos Pathogens.

Sincerely,

Lindsey Shallberg

Christopher Hunter

**Reviewer 1**

Major Issues: none

Minor Issues:

**Comment** Essentially all legends lack number of experimental replicates, some lack n numbers

**Response** We have updated the figure legends to make sure all contain numbers (N) in experimental groups. In addition, we now describe this in Material and Methods. For example, we have indicated in Figure 5 that the transcriptional profiling was performed on 4 pools of 2 mice each.

**Comment** Fig. 1E. Text and legend do not specify how OT-1 were initially activated pre-transfer

**Response** This was originally described in the Materials and Methods but the text and figure legends have been modified to clarify that OT-I were activated *in vitro* with agonist αCD3 and αCD28 antibodies for 48 hours and rested for 72 hours prior to transfer.

**Comment** Fig. 2D, 6C, 7D. Nur77+ and Nur77- rows are not specified

**Response** These UMAP figures (Fig 2D, 6C, and 7D) comprise analysis of the combined Nur77-GFP^-^ and Nur77-GFP^+^ OT-I populations. However, for each of these figures the separation of these populations based on expression of Nur77-GFP is shown (Fig 2C, 6B, and 7C). This allows readers to visualize how TCR engagement correlates with different populations. Consequently, we do not think there is a need to specify Nur77-GFP^+^ and Nur77-GFP^-^ rows.

**Comment** Fig 2E. Ki67 analysis is mentioned as part of figure 2E but no data are present in figure

**Response** Thank you for pointing out this discrepancy. These data were omitted from the figures but were not removed from the text. The text has been revised.

**Comment** Multi-panel figures seem to randomly assign letters and how many panels with different measurements are associated with a letter, without indicating the specifics in the legend. See 4 A and B as example

**Response** Thank you for pointing this out. The figure legends have been updated and expanded to clarify what the individual panels depict.

**Comment** Would be helpful for Fig 2 legend to include T. gondii challenge dose and route of administration, and whether the authors consider this a high-, moderate- or low-dose challenge, as this is quite relevant to how efficiently the transferred OT-1 would be stimulated.

**Response** This is a helpful suggestion. We have modified the legend as requested, but also the text has been modified to highlight that this combination of low parasite dose and small numbers of transferred OT-I (5,000) results in efficient T cell activation within 3 days.

**Comment** Source of the T. gondii is cited but would be helpful for the manuscript to briefly elaborate on the particulars of OVA expression so readers do not have to dig for it (eg, expressed off constitutive highly-expressed parasite promoter, OVA is secreted, whether OVA is efficiently presented from both tachyzoite and bradyzoite stage parasites seems to be unknown?)

**Response** Thank you for bringing up this point. A more extensive description of the parasites has been added to the text for clarity. Additionally, while OVA is driven from a constitutive promoter expressed in both the tachyzoite and bradyzoite stages, it is not currently clear whether CD8^+^ T cells can recognize bradyzoites. Please note that we highlight this knowledge gap in the final paragraph of the Discussion.

**Comment** Fig 2D: Perhaps a better color scale for relative expression could be chosen? Very minimal cells fall outside the middle 50% range of the scale as currently shown.

**Response** Thank you for this suggestion. One point of these plots is that these are activated T cells that express similar levels of certain activation markers but there are a subset of markers (CX3CR1, Bcl-2, PD-1, CD25, and TIM3) where sub-populations clearly fall outside the 50% range. While we are able to change the range of the scale (i.e. higher expression of the marker would have a more blue color) we do not want to misrepresent the expression patten of these markers on the OT-I. For example, Ly6C does not have much blue shading, but this is because almost all OT-I are uniformly positive for Ly6C (see below Ly6C expression on OT-I from infected spleens versus naive CD8^+^ T cells and Ly6C FMO).

**Comment** Fig 4E: Would be helpful to show the CD45.1 stain as well to demonstrate all OT-1 vs only Nur77-GFP+ (probably accidental omission as the legend describes these cells as appearing in white)

**Response** Thank you for pointing this out. There was an omission of CD45.1 in the figure description but this has been updated. Below you can see how we have classified Nur77-GFP^+^ OT-I, with the red circle indicates a Nur77-GFP^+^ OT-I, the purple circle indicates a Nur77-GFP^-^ OT-I, and the blue circle indicates background autofluorescence in the GFP channel.

**Comment** Fig 4E: Have the authors done any staining for tachyzoite vs bradyzoite markers to determine whether the parasites labeled as tachyzoites show any evidence of differentiation into bradyzoite stage? What infection time-point are the shown images from?

**Response** Good question. To be clear, we are not using IHC for parasites, rather we are detecting parasite expression of tdTomato which does not allow us to distinguish tachyzoites and bradyzoites. However, morphology of cysts (that contain bradyzoites) is distinct and forms the basis for the quantification at 28 dpi shown in Figure 4.

**Comment** Sulfadiazine is only active against tachyzoite-stage parasites. Would be helpful for authors to clarify whether they believe robust tachyzoite proliferation is still occurring at d21pi that is being targeted, or it is minimizing rare breakthroughs into tachyzoite stage? Also, does sulfadiazine cross the BBB where it would be able to act against CNS cysts?

**Response** Thanks for this question. It is exceedingly difficult to distinguish ongoing tachyzoite replication versus tachyzoites that result from cyst reactivation. Sulfadiazine does cross the BBB but is not active against bradyzoites (Ferreira et al, *Experimental Parasitology* 2006) and we now cite this study. Indeed, as seen in Figure 8B sulfadiazine treated mice had a lower tachyzoite parasite burden in leukocytes taken from the brains but this treatment does not distinguish the effects of sulphadiazine on parasites that were continuously replicating versus those that were derived from cyst reactivation. However, there is evidence of continuous levels of tachyzoite proliferation at 21 dpi and we cite this previous work on page 11. In addition, in ongoing studies we have used BFD1 KO parasites (that do not form cysts) and there is significant tachyzoite replication in the CNS. These studies are ongoing and these data are provided here only for the sake of the reviewer.

**Comment** Fig 7F: Authors should acknowledge that Landrith et al 2017 have also produced congruent data indicating that TRM populations can produce IFNg and TNF during chronic TE.

**Response** Thank you for the suggestion. Landrith et al, 2017 is cited in the manuscript multiple times, and this is the report that first described Trm formation in the CNS following *T. gondii* infection. We have now highlighted in the Results and Discussion section that they showed that these cells can produce IFN-γ and TNF-α.

**Reviewer 2**

Major Issues:

**Comment** The data in Figure 1 does an outstanding job of validating the model of OVA-specific Nur77-GFP expression. Although not relevant to the goals of this paper, it still might be of interest to some readers as to whether OT-1-specific T cells can contribute to control of OVA-expressing parasite – this does not appear to be mentioned in the manuscript. While this is a really useful system, it has some limitations (see comments below)

**Response** Whether OT-I T cells control OVA-expressing parasites is an excellent point, and has been addressed previously by the lab (Wilson et al, *Immunity* 2009) which showed that addition of OT-I can lead to reduced parasite levels. This is now mentioned in the Introduction.

**Comment** Line 158. This sentence and other interpretations in the manuscript seem to imply that “initial priming (which presumably means activation of naïve T cells) occurs only during the very early stages of the infection. This is one of my major concerns on interpretation of the results – how do the authors know that naïve T cells are not continuously recruited into the response throughout the infection? (Perhaps this could be addressed by transfer of naïve transgenic T cells into WT mice?). This sentence also implies that only “initial priming” is being observed between days 10-60 – but couldn’t some T cells be receiving multiple TCR-engaging events during this time – and so the observations are not solely on initial priming.

**Response** Thank you for articulating this concern. We do know that if we add in naive OT-I to mice infected for more than 20 days that these cells can become primed and expand. However, in the system being used here we bypass this issue. Thus, we find that with the transfer of low numbers of OT-I T cells prior to infection with a low parasite challenge the vast majority of these cells are primed in the first few days and this previous work is now cited. Thus, at the earliest time point that we present data (10 dpi) in infected mice all OT-I have an activated phenotype (see Fig 3A). This is a strength of this model and should mitigate the concern that naive OT-I are getting primed at later time points and confounding the interpretation. We agree with the reviewer that these OT-I are receiving multiple TCR-engaging events after priming, and the observations we report in the manuscript are profiling the impact of these non-priming TCR events. The text has been altered in several places with additional citations to make this clear to readers.

**Comment** Line 176 the wording of the last phrase in this sentence is confusing – why would some cells be “less likely to receive TCR signals” – are they lacking TCRs or other activating molecules – might be more accurate as “… have not received TCR signals; do we know TCR expression on these cells?

**Response** Thanks for this comment. We have modified the text to clarify that a subpopulation of KLRG1^+^CX3CR1^+^ OT-I are overrepresented in the Nur77-GFP^-^ population. We do know that these OT-I are equally positive for TCR (by Va2 staining). This point has been clarified in the text.

**Comment** The organization of the paper was also a bit confusing – the authors start making conclusions about memory formation, etc. based on the composition of T cells in the spleen. Since they ultimately look at T cells at the site of parasite replication, etc (the brain) and then compare this back to the spleen, it would seem to make more sense to discuss these compartments early in the paper rather than making conclusions based on spleen cells only (line 192) in the early part of the manuscript.

**Response** We appreciate this suggestion and in early drafts had considered a similar option. However, multiple studies show that the formation of different memory cell subsets begins early during infection, not just at later stages or after an infection has been cleared (Chu, et al, *Immunity* 2016; Kaech, et al, *Nature Immunology* 2003). The impact that secondary TCR activation has on the formation of these subpopulations has not been well characterized, and is the point of these experiments. From our perspective, it did not make sense to compare these compartments early when parasite involvement in the CNS is minimal. In addition, it is difficult to compare the impact of TCR stimulation on memory formation using splenic populations from chronically infected mice as it is a challenge to get enough Nur77-GFP^+^ OT-I from the spleens of these mice as they have largely resolved infection in the periphery. We hope this response is clear and we will retain the current organization as it reflects the temporal nature of this infection.

Here we have grouped a series of four comments that deal with a related issue of whether there is antigen presentation in the CNS:

**Comment** The sentence beginning line 203 is confusing – and perhaps gets at one of my major concerns about the work overall. The authors imply that T cells are being activated by antigens presented on infected cells or other accessory cells in the brain – but I can’t find the evidence for that.

**Comment** Toxoplasma and toxoplasma antigens are likely not only being presented in the brain, even in chronic infection. The authors do a nice job of differentiating T cells in the bloodstream from those in the brain tissue. But I don’t believe they can determine where T cell activation is occurring; the authors seem to suggest that Nur77-gfp+ cells in the brain were activated in the brain. This is almost certainly not the case in the very early stages of the infection, and even later, couldn’t T cells activated in non-brain parenchyma sites of replication (including in endothelial cells) enter the brain as GFP+ cells? Related to this, the conclusion in line 320 does not appear to be firmly demonstrated in the studies. Likewise the statement beginning line 329.

**Comment** Lines 201-215 in general – it is difficult to follow and understand the implications. Further, I don’t see that the data in this paragraph supports the conclusions that “Together, these data sets highlight that as the infection transitions to the chronic stage in the CNS there are enhanced levels of TCR activation that occurs at the interface between the brain and the vascular system, and in the parenchyma TCR activity appears largely restricted to sites of parasite replication.” The data here provide only anecdotal documentation of activated T cells in the brain and no comparison between sites where there are cysts, tachyzoites, or neither.

**Comment** I’m concerned that the possibility that activation of T cells outside the brain is not considered e.g. Line 320” Thus, despite the CNS being a tissue canonically considered immune privileged there are robust levels of antigen presentation and T cell activation that is required for local pathogen control.”

**Response** We think the main concern articulated in these four comments revolve around whether the T cells present in the CNS received TCR signals in other sites, perhaps even initial priming at the later stage of infection and then migrated into the CNS versus receiving TCR signals directly within the CNS. We hope our earlier response above establishes why we this is unlikely to be a consequence of new priming (by day 10 all OT-I have activated phenotype). Convention might also suggest that effector cells that encounter antigen in peripheral tissues (say the muscle) would be retained locally rather than migrate to other sites.

Line 203 addressed OT-I entering the brain at a time when *T. gondii* is present in the vascular endothelium (Konradt et al, *Nature Microbiology* 2016). We concur with the reviewer that at this stage of infection (day 10) that the OT-I can receive TCR stimulation (for example at the vasculature) and then migrate into the CNS. That is the basis of Fig 4C and is a topic in the discussion. However, as the infection progresses, there are minimal levels of Nur77-GFP^+^ OT-I in the blood (Fig 4C) at a time when there are numerous Nur77-GFP^+^ OT-I in the parenchyma. These data do not readily support the idea effector OT-I are seeing antigen elsewhere, and then expressing Nur77-GFP^+^ as they circulate and then migrate into the brain.

The reviewer is right that we did not provide evidence for local antigen presentation. In the Introduction and Discussion we now describe previous studies that indicate that antigen presentation occurs within the CNS. These previous reports from multiple laboratories show:

I. Infection results in increased numbers of DC in the CNS, and CD11c^+^ cells in the brains of *PRU-OVA* infected mice stain positive for H2-Kb SIINFEKL complexes and activate OT-I *ex vivo* (Fischer et al, *Journal of Immunology* 2000, John et al, *Plos Pathogens* 2009)
II. CD8^+^ T cells in the CNS of *T. gondii* infected mice show behavior consistent with TCR activation, including stalling around *T. gondii* parasites (Schaeffer et al, *Journal of Immunology* 2009; John et al, *Plos Pathogens* 2009)
III. Tachyzoite infected neurons present antigen to CD8^+^ T cells in the CNS during *T. gondii* infection (Salvioni et al, *Cell Reports* 2019)

It is unfortunate that the data are described as anecdotal. Consistent with a literature that presentation of parasite derived antigens occurs in the CNS, we believe our data detecting Nur77-GFP by IHC, flow cytometry, and live imaging showing the close association of OTI with areas of parasite replication and stalling behavior, combined with the transcriptional profiling, are consistent with Nur77-GFP^+^ OT-I found in the CNS during later timepoints getting local TCR signals. Also, IFN-γ production is linked to TCR activation, and in Figure 7F we show IFN-γ production by Nur77-GFP^+^ OT-I in the CNS. Finally, the characterization of the Nur77-GFP OT-I system in Figure 1 shows a decrease in GFP expression after removal of TCR stimulation, however there are still OT-I that express high levels of Nur77-GFP in the brain parenchyma at all time points, indicating these cells have recently seen antigen.

While imaging of Nur77-GFP OT-I has allowed us to correlate antigen stimulation with the location of tachyzoites vs cysts, the question about whether cysts are recognized by CD8^+^ T cells is the subject of ongoing research. However, we did devote the last paragraph of the discussion to this topic.

**Comment** Line 289: “These data demonstrate that TCR stimulation contributes to the heterogeneity seen in the CD8+ T cell population, and there is still considerable variation in T cell phenotypes unexplained by recent TCR stimulation.” I think the first part of this sentence can be more clearly stated as that the T cell populations in the spleen and brain are (as expected) different likely because antigen is being encountered by T cells in the brain but not (or less frequently) by those in the spleen. It is not clear what the second part of this sentence is referring to – or what specific data indicate this.

**Response** Thank you for pointing out the lack of clarity in this sentence. We have updated the text to better state our conclusions. The point here is that there is still considerable heterogeneity in the OT-I that are Nur77-GFP negative.

**Comment** Line 338 I don’t think the data support the conclusion that “ the studies presented here revealed that the OT-I Trm-like cells received frequent TCR stimulation”. I can’t find any data supporting activation actually occurring in the brain, much less the frequency of that stimulation. I agree that this is a reasonable presumption, I’m just not sure the data support it.

**Response** Figure 8A compares the response of CNS OT-I and shows there is an enrichment of CD69^+^CD103^+^ cells in the Nur77-GFP^+^ fraction (20% in Nur77-GFP^-^ vs 50% in Nur77-GFP^+^). In addition, when mice are treated with sulfadiazine to limit tachyzoite replication the OT-I lose expression of Nur77-GFP but the Trm are not lost from the CNS. It is a feature of Trm that they are tissue resident and that their expression of CD69 (required for tissue retention) and CD103 (a component of the integrin complex aEb7) are two signals associated with tissue retention. Thus, at this timepoint (6 weeks post infection) it is unlikely that these Trm will have seen antigen elsewhere and then trafficked to the brain while retaining Nur77-GFP expression.

**Comment** The authors propose that recently activated T cells (Nur77-GFP+) are associated with sites of parasites replication in the brain (starting line 216) but there appears to be no real quantitation of this – just some anecdotal observations. So the conclusions to this point are not well supported.

**Response** While we can readily detect OT-I throughout the CNS it was a major challenge to find Nur77-GFP^+^ OT-I when not first identifying sites of *T. gondii* infection. However, please note that in Figure 4E, we show areas of parasite replication as well as large cysts within the CNS of infected mice. *We then did quantify (Fig 4E) the number of Nur77-GFP^+^ OT-I that surround those two contrasting sites of infection.* Thus, we are able to compare association of Nur77-GFP^+^ OT-I with either sites of replication or latent cysts. We have revised the manuscript to better state our findings and concluded that “TCR activity appears to be enhanced at sites of parasite replication versus surrounding cysts” due to us finding very few instances of Nur77-GFP^+^ OT-I surrounding cysts versus sites of replication.

**Comment** Line 264 is probably the most important conclusions from the data “These data indicate that TCR activation, rather than bystander activation, is the main stimulus required for effector function …”

**Response** Thank you for the comment.

Minor Issues:

**Comment** Line 24: This sentence is not clear as written – perhaps more effective if written as “However, both Nur77-GFP negative and positive OT-I from infected mice can form memory populations when transferred to naïve mice.”

**Response** This has been edited in the text for clarity.

**Comment** Is ova in this system known to be expressed in bradyzoites?

**Response** Yes, OVA is driven by a constitutive promoter (ptub1; Pepper et al, *Infection and Immunity* 2004). This has been clarified in the text.

**Comment** Fig 1D – thoughts about why OVA peptide induces a higher percentage but lower level of Nur-77 relative to CPS-OVA disconnect between activation and level of activation

**Response** Firstly, it is important to emphasize that these populations are largely similar but in the infected cultures the % and MFI of GFP expression is slightly higher. We do not have a ready explanation but this may either be a consequence of infected cells receiving microbial signals that enhance their ability to form more stable DC:T cell interactions that result in more sustained TCR signaling. When using peptide these microbial signals would be absent. As this is highly speculative we have elected not to comment on this modest difference.

**Comment** Line 225 – perhaps a word missing here “It was also notable that in this (movie??) OT-I T cells that were non-motile and had a rounded appearance (two behaviors associated with TCR engagement) could be visualized in proximity to a cyst.

**Response** Thank you for catching this. This has been clarified in the text.

**Comment** Line 250 “had greater abundance of transcripts” – maybe better as “had fewer transcripts”

**Response** Thank you for this suggestion. In this sentence we were attempting to describe the Nur77-GFP^-^ OT-I, versus only describing the data in terms of what the expression patterns were for Nur77-GFP^+^ OT-I. Thus, we have kept this description the same.

**Comment** Line 275 – this paragraph begins with the goal of comparing spleen to brain – and this line “One of the most notable differences…” might be interpreted to be making that comparison but I think it is comparing GFP+ to GFP- in the brain – if I am not mistaken – but this should be clarified.

**Response** We apologize for lack of clarity. This is referencing the difference between Nur77-GFP^-^ and Nur77-GFP^+^ OT-I from the brain. The text has been updated.

**Reviewer 3:**

Major Issues:

**Comment** The major issue with this manuscript is perhaps a semantic one that is based on a limitation within the field for how chronic infections in mice are defined relative to how they clinically present in humans. In humans, a chronic infection is largely asymptomatic with little to no evidence of active parasite replication or inflammation. The animal model used here, C57 mice infected with type II strain parasites for 6 weeks, will still have significant numbers of tachyzoites and unresolved inflammation in their brains. Furthermore the animals’ immune-privileged state is compromised as evidenced by disruption of the blood brain barrier (e.g. Estato et al 2018 American Journal of Pathology). As the authors note in the first sentence of their discussion of this submission when highlighting their earlier work on T-cell motility in brains of Toxoplasma infected mice as well as other parts of the discussion, these mice have toxoplasmic encephalitis, which by definition is not a feature of the chronic phase of the infection. On the other hand, understanding T-cell responses during this phase is critical and seeing the differences between Figures 6 and 7 are important and critical to disseminate to the field. On the other hand, data from the sulfadiazine-treated mice more likely resemble a “chronic” infection and are also significant. With that said, some investigators (including perhaps the authors) may not agree and that is fine. Respectfully, however, I think that some discussion of this issue is warranted.

**Response** Thank you for raising this point. It’s always interesting to hear how this model is viewed by others. Firstly, there are rare reports of TE in the immune competent host and chorioretinitis in the immune competent host is a common manifestation of this infection. Nevertheless, we agree that this mouse model does not recapitulate asymptomatic infection in a healthy human. However, this model has been used as a clinically relevant model to understand the pathogenesis of human disease and to develop better drug treatments. Here, it is utilized to study the basic mechanisms of how T cells function in the CNS and mediate control of an infection. The Introduction has been altered to reflect this.

We agree it is likely that the treatment of mice with sulfadiazine more accurately models the chronic human state, and was one of the motivating factors in performing this experiment. We have modified the discussion to highlight that at present it is not clear if there are parasite specific Trm in humans.

Minor Issues:

**Comment** 1. While I imagine a secreted OVA strain was used for these experiments, please specifically clarify this.

**Response** Yes, we have updated the text for clarity that this is a secreted OVA strain.

**Comment** 2. Lines 161-162: I think that the sentence “Numbers of OT-1….” can be removed?

**Response** Thank you for the suggestion, we have included this sentence to reference the overall kinetics of OT-I in the spleen throughout this time course.

**Comment** 3. In Fig. 1E, please clarify whether the T-cells were pre-activated with anti-CD3 or SIINFEKL peptide.

**Response** The OT-I were activated with αCD3 and αCD28, this has been updated in the text and figure legend.

**Comment** 4. In 1B, how long were cells stimulated before removal.

**Response** The OT-I were stimulated for 48 hours prior to removal of stimulation. This has been added to the text versus relying solely on the figure legend.

**Comment** 5. Please define the statistical significance for each of the asterisks shown in the figures.

**Response** Thank you for pointing this out. These have been added to figure legends.

**Comment** 6. Please provide white scale bars in Figure 4E. In addition, please indicate whether the images are from a single slice or maximal projections of a series of slices. Also, please indicate whether the smaller green spots are background or parts of parasites whose edges are located at the top or bottom of the slice/stack.

**Response** The scale bars were inadvertently cropped, they have been restored in the revised figures. The smaller green spots are likely background autofluorescence or background staining (as we need to stain for anti-GFP in these images due to fixation of the brains quenching GFP fluorescence), and we have seen this level of background in brains of mice that have not received transfers of Nur77-GFP OT-I as well as naive mice. GFP expression is only indicated where there is also membrane shaped staining for CD45.1, an obvious nucleus by DAPI staining, and intracellular GFP staining that overlays with DAPI inside the CD45.1^+^ membrane. For example, below the red circle indicates a GFP^+^ OT-I, the purple circle indicates a GFP^-^ OT-I, and the blue circle indicates background autofluorescence in the GFP channel.

**Comment** 7. Please provide more information regarding gating strategies for the FACS data. In addition, can the authors go back and check that the quadrants are defined correctly between Figures 3A and 3E because while the quadrants change rather dramatically, the position of the lower cell populations do not. This is important because it defines whether the cells are CX3CR1 positive or negative.

**Response** Please find the FMO gating for KLRG1 and CX3CR1 expression for Figures 3A and 3E below. These experiments were run on the cytometer at separate times, thus voltages were different causing different gating. These plots have been added to the supplemental figures to display accurate gating strategies for these populations.
