# Supplementary material for: Impact of secondary TCR engagement on the heterogeneity of pathogen-specific CD8+ T cell response during acute and chronic toxoplasmosis
Source: PLoS Pathog. 2022 Jun 21;18(6):e1010296. doi: 10.1371/journal.ppat.1010296 (PMC9249239; doi:10.1371/journal.ppat.1010296)
Supplement: Peer review history

# Peer review: Decision Letter 0

**DOI:** 10.1371/journal.ppat.1010296.r001

**Authors:** Eric Y Denkers, Dominique Soldati-Favre

7 Mar 2022

Dear Dr. Hunter,

Thank you very much for submitting your manuscript "Impact of secondary TCR engagement on the heterogeneity of pathogen-specific CD8+ T cell response during acute and chronic toxoplasmosis" for consideration at PLOS Pathogens. As with all papers reviewed by the journal, your manuscript was reviewed by members of the editorial board and by several independent reviewers. In light of the reviews (below this email), we would like to invite the resubmission of a significantly-revised version that takes into account the reviewers' comments.

We cannot make any decision about publication until we have seen the revised manuscript and your response to the reviewers' comments. Your revised manuscript is also likely to be sent to reviewers for further evaluation.

When you are ready to resubmit, please upload the following:

[1] A letter containing a detailed list of your responses to the review comments and a description of the changes you have made in the manuscript. Please note while forming your response, if your article is accepted, you may have the opportunity to make the peer review history publicly available. The record will include editor decision letters (with reviews) and your responses to reviewer comments. If eligible, we will contact you to opt in or out.

[2] Two versions of the revised manuscript: one with either highlights or tracked changes denoting where the text has been changed; the other a clean version (uploaded as the manuscript file).

Important additional instructions are given below your reviewer comments.

Please prepare and submit your revised manuscript within 60 days. If you anticipate any delay, please let us know the expected resubmission date by replying to this email. Please note that revised manuscripts received after the 60-day due date may require evaluation and peer review similar to newly submitted manuscripts.

Thank you again for your submission. We hope that our editorial process has been constructive so far, and we welcome your feedback at any time. Please don't hesitate to contact us if you have any questions or comments.

Sincerely,

Eric Y Denkers

Associate Editor

PLOS Pathogens

Dominique Soldati-Favre

Section Editor

PLOS Pathogens

Kasturi Haldar

Editor-in-Chief

PLOS Pathogens

​orcid.org/0000-0001-5065-158X

Michael Malim

Editor-in-Chief

PLOS Pathogens

orcid.org/0000-0002-7699-2064

***********************

Reviewer's Responses to Questions

**Part I - Summary**

Please use this section to discuss strengths/weaknesses of study, novelty/significance, general execution and scholarship.

Reviewer #1: Shallberg et al

Major Findings & Strengths

-Effector and memory CD8 T-cells are well-established important mediators of control of intracellular infections. Antigen engagement of CD8 T-cell TCR’s prompt expansion and subsequent generation of memory populations. How antigen re-engagement of TCR’s precisely influences the generation and maintenance of memory CD8 T-cells remains unclear, particularly for chronic infection models. This manuscript uses a murine model of T. gondii infection to interrogate this question. By pairing transgenic OVA-expressing T. gondii parasites with Nur77-GFP reporter OT-1 cells (transgenic CD8 specific to OVA peptide), this manuscript demonstrates that recent TCR engagement markedly influences gene expression and modestly changes the phenotype of effector CD8 T cells populations (as expected), but does not markedly influence their subsequent ability to form memory populations after sorting and transfer. The reason(s) for this are unclear. The authors also address the gene expression, phenotype and movement of Nur77+ and Nur77- OT-I in the brain, again with largely expected alterations in cells that have recently encountered antigen.

-The manuscript in general and the introduction in particular is very clearly and articulately written.

-Although the conceptual advance is modest, these are novel findings for the T. gondii model, the experimental findings are generally well supported and seem appropriate in scope for PLOS Pathogens.

Weaknesses & Limitations

-The authors allude to addressing the topic of how chronic T. gondii infection alters the memory CD8 T response. However, no data are presented whether the OVA produced from the transgenic T. gondii-OVA parasites is robustly generated and immunostimulatory from parasites that have differentiated into the chronic bradyzoite stage. Accordingly, it remains unclear whether the studied TCR activation events are reflective of rare events of parasites breaking through into the proliferative tachyzoite form, or truly reflective of the chronic bradyzoite stage. Further work addressing this shortcoming is highlighted at the end of the discussion, but this limitation should be also acknowledged in the introduction and descriptions of the experimental approach.

Reviewer #2: This study reports on the development and validation of a nice model for the accessing TCR engagement in vivo and in vitro and uses high parameter flow cytometry and robust transcriptional analysis to attempt to monitor the impact of that engagement, all in a model of T. gondii infection in mice. These are powerful tools and the investigators employ them well. The data appear solid. However, I am concerned that the goals of the study remain beyond the reach of this research approach and that a number of the conclusions seem to be obvious from many previous studies (e.g. line 174” …recent TCR activation is not closely associated with those T cells that express markers of an effector population…”), while other conclusions, while consistent with the observations, are not proven by them. Also, the overall message from the study is not clear – presumably this is what is indicated in the final sentence of the abstract. One would expect that T cells at different times post-activation would be phenotypically heterogeneous – this is fairly well known. The “this process” referred to in this sentence must refer to “recent TCR activation”. It is not clear to me what data shows that recent TCR activation has a limited impact on memory populations. Perhaps I am missing it, but I just don’t get the conclusion (and new insights) that can be drawn from the study as presented.

Reviewer #3: This is an important paper examining the phenotype of CD8 T-cells upon antigen restimulation in brains of Toxoplasma infected mice. Although descriptive in nature, the work represents an important first step in this area and is therefore is appropriate for PLoS Pathogens and of interest to its readers. In this reviewer’s opinion, no additional experiments are needed – they represent a complete story and are well performed with rigorous controls. In addition, the paper is very clear and well-written. But I do have one major comment that should be considered and/or addressed before publication as well as some other minor points for clarification.

**********

**Part II – Major Issues: Key Experiments Required for Acceptance**

Please use this section to detail the key new experiments or modifications of existing experiments that should be absolutely required to validate study conclusions.

Generally, there should be no more than 3 such required experiments or major modifications for a "Major Revision" recommendation. If more than 3 experiments are necessary to validate the study conclusions, then you are encouraged to recommend "Reject".

Reviewer #1: (No Response)

Reviewer #2: The data in Figure 1 does an outstanding job of validating the model of OVA-specific Nur77-GFP expression. Although not relevant to the goals of this paper, it still might be of interest to some readers as to whether OT-1-specific T cells can contribute to control of OVA-expressing parasite – this does not appear to be mentioned in the manuscript. While this is a really useful system, it has some limitations (see comments below)

Line 158. This sentence and other interpretations in the manuscript seem to imply that “initial priming (which presumably means activation of naïve T cells) occurs only during the very early stages of the infection. This is one of my major concerns on interpretation of the results – how do the authors know that naïve T cells are not continuously recruited into the response throughout the infection? (Perhaps this could be addressed by transfer of naïve transgenic T cells into WT mice?). This sentence also implies that only “initial priming” is being observed between days 10-60 – but couldn’t some T cells be receiving multiple TCR-engaging events during this time – and so the observations are not solely on initial priming.

Line 176 the wording of the last phrase in this sentence is confusing – why would some cells be “less likely to receive TCR signals” – are they lacking TCRs or other activating molecules – might be more accurate as “… have not received TCR signals; do we know TCR expression on these cells?

The organization of the paper was also a bit confusing – the authors start making conclusions about memory formation, etc. based on the composition of T cells in the spleen. Since they ultimately look at T cells at the site of parasite replication, etc (the brain) and then compare this back to the spleen, it would seem to make more sense to discuss these compartments early in the paper rather than making conclusions based on spleen cells only (line 192) in the early part of the manuscript.

The sentence beginning line 203 is confusing – and perhaps gets at one of my major concerns about the work overall. The authors imply that T cells are being activated by antigens presented on infected cells or other accessory cells in the brain – but I can’t find the evidence for that. Toxoplasma and toxoplasma antigens are likely not only being presented in the brain, even in chronic infection. The authors do a nice job of differentiating T cells in the bloodstream from those in the brain tissue. But I don’t believe they can determine where T cell activation is occurring; the authors seem to suggest that Nur77-gfp+ cells in the brain were activated in the brain. This is almost certainly not the case in the very early stages of the infection, and even later, couldn’t T cells activated in non-brain parenchyma sites of replication (including in endothelial cells) enter the brain as GFP+ cells? Related to this, the conclusion in line 320 does not appear to be firmly demonstrated in the studies. Likewise the statement beginning line 329.

Lines 201-215 in general – it is difficult to follow and understand the implications. Further, I don’t see that the data in this paragraph supports the conclusions that “Together, these data sets highlight that as the infection transitions to the chronic stage in the CNS there are enhanced levels of TCR activation that occurs at the interface between the brain and the vascular system, and in the parenchyma TCR activity appears largely restricted to sites of parasite replication.” The data here provide only anecdotal documentation of activated T cells in the brain and no comparison between sites where there are cysts, tachyzoites, or neither.

Line 289: “These data demonstrate that TCR stimulation contributes to the heterogeneity seen in the CD8+ T cell population, and there is still considerable variation in T cell phenotypes unexplained by recent TCR stimulation.” I think the first part of this sentence can be more clearly stated as that the T cell populations in the spleen and brain are (as expected) different likely because antigen is being encountered by T cells in the brain but not (or less frequently) by those in the spleen. It is not clear what the second part of this sentence is referring to – or what specific data indicate this.

I’m concerned that the possibility that activation of T cells outside the brain is not considered e.g. Line 320” Thus, despite the CNS being a tissue canonically considered immune privileged there are robust levels of antigen presentation and T cell activation that is required for local pathogen control.”

Line 338 I don’t think the data support the conclusion that “ the studies presented here revealed that the OT-I Trm-like cells received frequent TCR stimulation”. I can’t find any data supporting activation actually occurring in the brain, much less the frequency of that stimulation. I agree that this is a reasonable presumption, I’m just not sure the data support it.

The authors propose that recently activated T cells (Nur77-GFP+) are associated with sites of parasites replication in the brain (starting line 216) but there appears to be no real quantitation of this – just some anecdotal observations. So the conclusions to this point are not well supported.

Line 264 is probably the most important conclusions from the data “These data indicate that TCR activation, rather than bystander activation, is the main stimulus required for effector function …”

Reviewer #3: The major issue with this manuscript is perhaps a semantic one that is based on a limitation within the field for how chronic infections in mice are defined relative to how they clinically present in humans. In humans, a chronic infection is largely asymptomatic with little to no evidence of active parasite replication or inflammation. The animal model used here, C57 mice infected with type II strain parasites for 6 weeks, will still have significant numbers of tachyzoites and unresolved inflammation in their brains. Furthermore the animals’ immune-privileged state is compromised as evidenced by disruption of the blood brain barrier (e.g. Estato et al 2018 American Journal of Pathology). As the authors note in the first sentence of their discussion of this submission when highlighting their earlier work on T-cell motility in brains of Toxoplasma infected mice as well as other parts of the discussion, these mice have toxoplasmic encephalitis, which by definition is not a feature of the chronic phase of the infection. On the other hand, understanding T-cell responses during this phase is critical and seeing the differences between Figures 6 and 7 are important and critical to disseminate to the field. On the other hand, data from the sulfadiazine-treated mice more likely resemble a “chronic” infection and are also significant. With that said, some investigators (including perhaps the authors) may not agree and that is fine. Respectfully, however, I think that some discussion of this issue is warranted.

**********

**Part III – Minor Issues: Editorial and Data Presentation Modifications**

Please use this section for editorial suggestions as well as relatively minor modifications of existing data that would enhance clarity.

Reviewer #1: General concerns

I found the legends inadequate and some figures lack critical specifying information

Essentially all legends lack number of experimental replicates, some lack n numbers

Fig. 1E. Text and legend do not specify how OT-1 were initially activated pre-transfer

Fig. 2D, 6C, 7D. Nur77+ and Nur77- rows are not specified

Fig 2E. Ki67 analysis is mentioned as part of figure 2E but no data are present in figure

Multi-panel figures seem to randomly assign letters and how many panels with different measurements are associated with a letter, without indicating the specifics in the legend. See 4 A and B as example

Would be helpful for Fig 2 legend to include T. gondii challenge dose and route of administration, and whether the authors consider this a high-, moderate- or low-dose challenge, as this is quite relevant to how efficiently the transferred OT-1 would be stimulated.

-Source of the T. gondii is cited but would be helpful for the manuscript to briefly elaborate on the particulars of OVA expression so readers do not have to dig for it (eg, expressed off constitutive highly-expressed parasite promoter, OVA is secreted, whether OVA is efficiently presented from both tachyzoite and bradyzoite stage parasites seems to be unknown?)

-Fig 2D: Perhaps a better color scale for relative expression could be chosen? Very minimal cells fall outside the middle 50% range of the scale as currently shown.

-Fig 4E: Would be helpful to show the CD45.1 stain as well to demonstrate all OT-1 vs only Nur77-GFP+ (probably accidental omission as the legend describes these cells as appearing in white)

-Fig 4E: Have the authors done any staining for tachyzoite vs bradyzoite markers to determine whether the parasites labeled as tachyzoites show any evidence of differentiation into bradyzoite stage? What infection time-point are the shown images from?

-Sulfadiazine is only active against tachyzoite-stage parasites. Would be helpful for authors to clarify whether they believe robust tachyzoite proliferation is still occurring at d21pi that is being targeted, or it is minimizing rare breakthroughs into tachyzoite stage? Also, does sulfadiazine cross the BBB where it would be able to act against CNS cysts?

Fig 7F: Authors should acknowledge that Landrith et al 2017 have also produced congruent data indicating that TRM populations can produce IFNg and TNF during chronic TE.

Reviewer #2: Line 24: This sentence is not clear as written – perhaps more effective if written as “However, both Nur77-GFP negative and positive OT-I from infected mice can form memory populations when transferred to naïve mice.”

Is ova in this system known to be expressed in bradyzoites?

Fig 1D – thoughts about why OVA peptide induces a higher percentage but lower level of Nur-77 relative to CPS-OVA disconnect between activation and lelve of activation

Line 225 – perhaps a word missing here “It was also notable that in this (movie??) OT-I T cells that were non-motile and had a rounded appearance (two behaviors associated with TCR engagement) could be visualized in proximity to a cyst.

Line 250 “had greater abundance of transcripts” – maybe better as “had fewer transcripts”

Line 275 – this paragraph begins with the goal of comparing spleen to brain – and this line “One of the most notable differences…” might be interpreted to be making that comparison but I think it is comparing GFP+ to GFP- in the brain – if I am not mistaken – but this should be clarified.

Reviewer #3: Minor comments.

1. While I imagine a secreted OVA strain was used for these experiments, please specifically clarify this.

2. Lines 161-162: I think that the sentence “Numbers of OT-1….” can be removed?

3. In Fig. 1E, please clarify whether the T-cells were pre-activated with anti-CD3 or SIINFEKL peptide.

4. In 1B, how long were cells stimulated before removal.

5. Please define the statistical significance for each of the asterisks shown in the figures.

6. Please provide white scale bars in Figure 4E. In addition, please indicate whether the images are from a single slice or maximal projections of a series of slices. Also, please indicate whether the smaller green spots are background or parts of parasites whose edges are located at the top or bottom of the slice/stack.

7. Please provide more information regarding gating strategies for the FACS data. In addition, can the authors go back and check that the quadrants are defined correctly between Figures 3A and 3E because while the quadrants change rather dramatically, the position of the lower cell populations do not. This is important because it defines whether the cells are CX3CR1 positive or negative.

**********

PLOS authors have the option to publish the peer review history of their article (what does this mean?). If published, this will include your full peer review and any attached files.

If you choose “no”, your identity will remain anonymous but your review may still be made public.

**Do you want your identity to be public for this peer review?** For information about this choice, including consent withdrawal, please see our Privacy Policy.

Reviewer #1: No

Reviewer #2: No

Reviewer #3: No

Figure Files:

While revising your submission, please upload your figure files to the Preflight Analysis and Conversion Engine (PACE) digital diagnostic tool, https://pacev2.apexcovantage.com. PACE helps ensure that figures meet PLOS requirements. To use PACE, you must first register as a user. Then, login and navigate to the UPLOAD tab, where you will find detailed instructions on how to use the tool. If you encounter any issues or have any questions when using PACE, please email us at figures@plos.org.

Data Requirements:

Please note that, as a condition of publication, PLOS' data policy requires that you make available all data used to draw the conclusions outlined in your manuscript. Data must be deposited in an appropriate repository, included within the body of the manuscript, or uploaded as supporting information. This includes all numerical values that were used to generate graphs, histograms etc.. For an example see here on PLOS Biology: http://www.plosbiology.org/article/info%3Adoi%2F10.1371%2Fjournal.pbio.1001908#s5.

Reproducibility:

To enhance the reproducibility of your results, we recommend that you deposit your laboratory protocols in protocols.io, where a protocol can be assigned its own identifier (DOI) such that it can be cited independently in the future. Additionally, PLOS ONE offers an option to publish peer-reviewed clinical study protocols. Read more information on sharing protocols at https://plos.org/protocols?utm_medium=editorial-email&utm_source=authorletters&utm_campaign=protocols

# Author comment: Author response to Decision Letter 0

**DOI:** 10.1371/journal.ppat.1010296.r002

6 Apr 2022

Attachment Submitted filename: Reviewer_response.docx Click here for additional data file.

# Peer review: Decision Letter 1

**DOI:** 10.1371/journal.ppat.1010296.r003

**Authors:** Eric Y Denkers, Dominique Soldati-Favre

6 May 2022

Dear Dr. Hunter,

We are pleased to inform you that your manuscript 'Impact of secondary TCR engagement on the heterogeneity of pathogen-specific CD8+ T cell response during acute and chronic toxoplasmosis' has been provisionally accepted for publication in PLOS Pathogens.

Before your manuscript can be formally accepted you will need to complete some formatting changes, which you will receive in a follow up email. A member of our team will be in touch with a set of requests.

Please note that your manuscript will not be scheduled for publication until you have made the required changes, so a swift response is appreciated.

IMPORTANT: The editorial review process is now complete. PLOS will only permit corrections to spelling, formatting or significant scientific errors from this point onwards. Requests for major changes, or any which affect the scientific understanding of your work, will cause delays to the publication date of your manuscript.

Should you, your institution's press office or the journal office choose to press release your paper, you will automatically be opted out of early publication. We ask that you notify us now if you or your institution is planning to press release the article. All press must be co-ordinated with PLOS.

Thank you again for supporting Open Access publishing; we are looking forward to publishing your work in PLOS Pathogens.

Best regards,

Eric Y Denkers

Associate Editor

PLOS Pathogens

Dominique Soldati-Favre

Section Editor

PLOS Pathogens

Kasturi Haldar

Editor-in-Chief

PLOS Pathogens

​orcid.org/0000-0001-5065-158X

Michael Malim

Editor-in-Chief

PLOS Pathogens

orcid.org/0000-0002-7699-2064

***********************************************************

Reviewer Comments (if any, and for reference):

Reviewer's Responses to Questions

**Part I - Summary**

Please use this section to discuss strengths/weaknesses of study, novelty/significance, general execution and scholarship.

Reviewer #1: The authors responded satisfactorily to my minor comments and the manuscript has improved sufficiently to warrant publication

Reviewer #2: (No Response)

**********

**Part II – Major Issues: Key Experiments Required for Acceptance**

Please use this section to detail the key new experiments or modifications of existing experiments that should be absolutely required to validate study conclusions.

Generally, there should be no more than 3 such required experiments or major modifications for a "Major Revision" recommendation. If more than 3 experiments are necessary to validate the study conclusions, then you are encouraged to recommend "Reject".

Reviewer #1: (No Response)

Reviewer #2: (No Response)

**********

**Part III – Minor Issues: Editorial and Data Presentation Modifications**

Please use this section for editorial suggestions as well as relatively minor modifications of existing data that would enhance clarity.

Reviewer #1: (No Response)

Reviewer #2: (No Response)

**********

PLOS authors have the option to publish the peer review history of their article (what does this mean?). If published, this will include your full peer review and any attached files.

If you choose “no”, your identity will remain anonymous but your review may still be made public.

**Do you want your identity to be public for this peer review?** For information about this choice, including consent withdrawal, please see our Privacy Policy.

Reviewer #1: No

Reviewer #2: No

# Editor report: Acceptance letter

**DOI:** 10.1371/journal.ppat.1010296.r004

**Authors:** Eric Y Denkers, Dominique Soldati-Favre

15 Jun 2022

Dear Dr. Hunter,

We are delighted to inform you that your manuscript, "Impact of secondary TCR engagement on the heterogeneity of pathogen-specific CD8+ T cell response during acute and chronic toxoplasmosis," has been formally accepted for publication in PLOS Pathogens.

We have now passed your article onto the PLOS Production Department who will complete the rest of the pre-publication process. All authors will receive a confirmation email upon publication.

The corresponding author will soon be receiving a typeset proof for review, to ensure errors have not been introduced during production. Please review the PDF proof of your manuscript carefully, as this is the last chance to correct any scientific or type-setting errors. Please note that major changes, or those which affect the scientific understanding of the work, will likely cause delays to the publication date of your manuscript. Note: Proofs for Front Matter articles (Pearls, Reviews, Opinions, etc...) are generated on a different schedule and may not be made available as quickly.

Soon after your final files are uploaded, the early version of your manuscript, if you opted to have an early version of your article, will be published online. The date of the early version will be your article's publication date. The final article will be published to the same URL, and all versions of the paper will be accessible to readers.

Thank you again for supporting open-access publishing; we are looking forward to publishing your work in PLOS Pathogens. 

Best regards,

Kasturi Haldar

Editor-in-Chief

PLOS Pathogens

​orcid.org/0000-0001-5065-158X

Michael Malim

Editor-in-Chief

PLOS Pathogens

orcid.org/0000-0002-7699-2064
